# Anti-NMDAR-Positive Small-Cell Lung Cancer Paraneoplastic Limbic Encephalitis: A Case Report and Literature Review

**DOI:** 10.1155/2020/5269352

**Published:** 2020-06-02

**Authors:** Raman Sohal, Steven H. Adams, Vishal Phogat, Abha Harish, Carlos Ynigo D. Lopez, Michael P. A. Williams, Kamal K. Khurana, Basel Abuzuaiter, Nia Jagroop, Bhavya Narapureddy

**Affiliations:** ^1^Department of Medicine, SUNY Upstate Medical University Hospital, 750 E Adams St., Syracuse, NY 13210, USA; ^2^Department of Pathology, SUNY Upstate Medical University Hospital, 750 E Adams St., Syracuse, NY 13210, USA; ^3^Department of Neurology, SUNY Upstate Medical University Hospital, 750 E Adams St., Syracuse, NY 13210, USA

## Abstract

**Introduction:**

Paraneoplastic limbic encephalitis (PLE) is a rare disease that presents as rapid onset dementia characterized by short-term memory loss (STM), anxiety, and behavioral changes. Anti-NMDAR antibodies are unfrequently reported in PLE associated with small-cell lung cancer (SCLC). Given that PLE can precede the diagnosis of cancer, it is very important that once infectious, metabolic, nutritional, or structural disorders associated with short-term memory loss are ruled out that vigorous effort must be made to rule out underlying malignancy.

**Case:**

We report a rare case of PLE as the presenting symptom of SCLC. A 72-year-old male with history of COPD was brought to the ED by his wife after he was found to have short-term memory loss, including forgetfulness of his wedding anniversary the day before, and anxiety. Neurological exam showed impaired short-term recall on MOCA. CT head showed no evidence of infarct. Lumbar puncture was performed which showed lymphocytic pleocytosis, a nonspecific inflammatory change. CSF panel was negative for HSV, *Neisseria*, *Hemophilus*, *E. coli*, and HIV. Initial EEG was unremarkable, though a repeat EEG showed mild slowing of the posterior dominant rhythm consistent with mild encephalopathy. MRI showed equivocal increased FLAIR on T2-weighted images in the bilateral temporal lobes, left greater than right. CTA thorax showed bulky mediastinal and right hilar LAD. FNA of the R4 lymph node revealed SCLC. The NM bone scan showed no osteoblastic lesions. While the serum autoantibody panel was positive for anti-NMDAR, the CSF autoantibody panel returned entirely negative. Chemotherapy with etoposide and cisplatin was started on Day 4 of admission. The patient's neurological symptoms showed improvement following chemotherapy.

**Conclusion:**

This case highlights the importance of recognizing short-term memory loss as a feature of PLE.

## 1. Case

Our patient is a 72-year-old Caucasian right-handed male with a past medical history significant for hypertension, hypercholesterolemia, COPD, coronary artery disease, and status post a recent umbilical hernia repair, admitted for short-term memory loss that began acutely on a mid-September morning (Day 1). The patient arrived anxious, worried that he was losing his memory. As per the patient's wife, he was feeling well, driving, and behaving normally on the previous day. He had gone to sleep at approximately 21:00 in his normal state of health but awoke the following morning with inability to recall events, including their 50th wedding anniversary party, of the prior day. His wife denied any symptoms of behavioral or personality changes in her husband prior to that ominous morning. The patient had a 50-pack-year smoking history, quitting 2.5 years prior to presentation, but was without any illicit drug or significant alcohol history. The patient's review of systems was positive for cough, congestion, anxiety, confusion, dizziness, and light-headedness only, denying any chest pain, shortness of breath, abdominal pain, loss of consciousness, history of recent falls or injury, weakness, numbness, tingling, speech difficulty, slurred speech, vision changes, headache, vertigo, photophobia, neck pain, nausea, or vomiting.

His vitals were within normal limits, with the exception of his blood pressure which measured 153/78 mm Hg (supine). On neurological examination, the patient was alert and oriented to person, place, month, and year. He was able to follow complex commands crossing his body's midline. On memory span testing, he recalled all three words accurately and immediately with categorical prompts. On the digit span test, he was able to recall five digits forward and four in reverse. Abstraction was preserved. He was without aphasia. Serial 7's were intact, and he was able to spell “world” forward and backwards. He was able to name all his children's names and their spouses but had difficulty with some of his grandchildren's names. He had only 1/3 object recall at three minutes, prompting with these categories did not improve his recall. His cranial nerve, sensory, and motor exams, and cerebellar tests for coordination and gait, were unremarkable. The remainder of the physical exam was normal. His CBC, BMP, LFT, and ammonia values were within normal limits. Ethyl alcohol tested negative. Serum thyroglobulin (0.9 IU/mL) and thyroperoxidase antibody (0.4 IU/mL) counts were normal; however, serum thyroglobulin antibody was elevated at 24.5 IU/mL.

**Figure 1:**
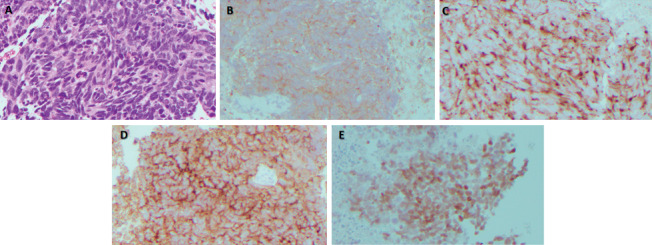
Microscopy (slides prepared for cell block analysis): (a) hematoxylin and eosin (H&E) slides consists of malignant cells, single and molding in groups with increased nuclear to cytoplasmic ratios, irregular nuclear contours, and hyperchromatic (200x magnification). ((b)–(e)) Immunohistochemistry (IHC) stains of synaptophysin (b), chromogranin (c), CD56 (d), and TTF-1 (e) are positive in the malignant cells (200x magnification). P63 IHC stain is negative (not shown).

Routine EEG was within range of normal variation. Noncontrast CT head was insignificant for any acute ischemic or hemorrhagic event. MRI brain with and without gadolinium showed no evidence of edema, mass effect, or metastasis, although patchy areas of T2 prolongation in the subcortical and periventricular white matter were noted. These were initially attributed to moderate chronic microvascular white matter ischemic changes in light of the patient's chronological age. Because his chest X-rays showed a widened mediastinum with apparent mass effect and tracheal deviation to the left, a CTA thorax was obtained, which showed bulky mediastinal and right hilar lymphadenopathy concerning for malignancy. Fine-needle aspiration of the R4 lymph nodes was positive for small-cell carcinoma (Figure 1).

**Figure 2:**
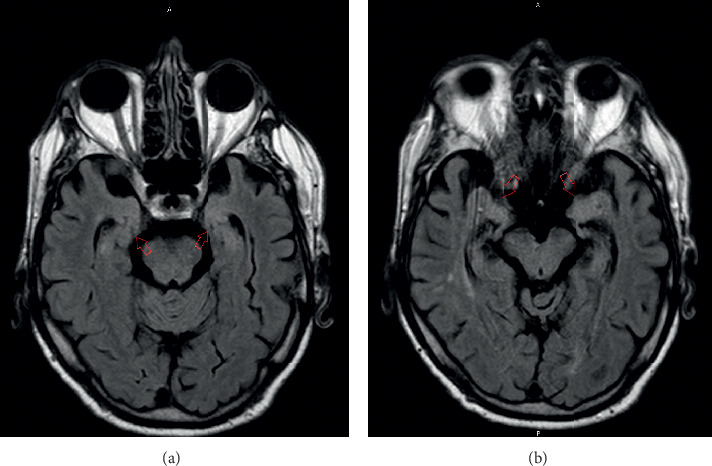
((a) and (b)) Axial FLAIR MRI showing slight increased signal in bilateral temporal lobes.

At this point, the patient's neurological symptoms were considered to be of possible paraneoplastic etiology. With this new consideration in mind, the MRI brain findings of bilateral (left more than right) medial temporal lobe hyperintensity compared with the rest of the temporal lobes were reassessed by the neurology service as possible manifestations of PLE. However, the neuroradiology service, upon reviewing the brain MRI, was not convinced that the findings represented limbic encephalitis (Figure 2).

Cerebrospinal fluid tests were ordered: protein was WNL at 34 mg/dl and glucose was WNL at 62. CSF was colorless and clear, with 14 RBCs and 6 total nucleated cells. Lymphocytic CSF pleocytosis was detected with 93% lymphocytes and 7% monocytes/macrophages. CSF culture and stain showed 3+ WBCs (WNL) with no organism growth. CSF pathogen panel PCR was negative for the 14 common pathogens responsible for community-acquired meningitis or encephalitis including viruses, bacteria, and yeast. CSF cytology showed no evidence of malignancy. Thus, both MRI and CSF cytology confirmed no metastasis to the brain.

On Day 3 of hospitalization, his MOCA score was 21/30 with deficits in delayed recall, visuospatial ability, and orientation. NM bone scan whole body imaging showed no evidence of osteoblastic metastatic bone disease.

On Day 4, chemotherapy was initiated and the patient received three rounds (a 3-day treatment regimen) of cisplatin/etoposide.

On Day 5, neurological improvement was noted on the second day of cycle 1 of chemotherapy, with the patient able to complete the visuospatial part of MOCA that he had missed previously, although he still had impaired delayed recall. The patient did receive 1 g of solumedrol before deciding with the neurology team to not continue with an IV steroid course because he was improving with chemotherapy. He was discharged after three days of cycle 1 of chemotherapy with encouraging signs of neurological improvement. Radiation oncology was also consulted to initiate radiation therapy as outpatient with a plan for radiation to begin with cycle 2 of chemotherapy.

On Day 13, 18F-FDG PET/CT imaging showed right upper lobe metabolically active lung mass compatible with a lung primary, and right hilar and mediastinal lymphadenopathy compatible with metastatic disease. Otherwise, no metastatic disease was detected. His SCLC was described as cT1cN2M0, stage IIIA. His forgetfulness showed slight improvement.

On Day 18, a serum encephalopathy autoantibody panel returned positive for NMDAR Ab (CBA). Anti-NMDAR antibody was detected when testing at 1 : 10 dilution by a cell-based assay only. Higher titers of antibody were not detected by reflex testing at 1:120 dilution by a tissue-based assay. Samples were sent to Mayo Clinic Laboratories, Rochester, for encephalopathy, autoimmune evaluation, and serum (Test ID: ENS2) which tests for AChR ganglionic neuronal Ab, S; AMPA-R Ab CBA, S; amphiphysin Ab, S; anti-glial nuclear Ab, type 1; anti-neuronal nuclear Ab, type 1; anti-neuronal nuclear Ab, type 2; anti-neuronal nuclear Ab, type 3; CASPR2-IgG CBA, S; CRMP-5-IgG, S; DPPX Ab IFA, S; GABA-B-R Ab CBA, S; GAD65 Ab assay, S; GFAP IFA, S; LGI1-IgG CBA, S; mGluR1 Ab IFA, S; NMDAR Ab CBA, S; N-type calcium channel Ab; P/Q-type calcium channel Ab; Purkinje cell cytoplasmic Ab type 1; Purkinje purkinje cell cytoplasmic Ab type 2; Purkinje cell cytoplasmic Ab type Tr.

On Day 26, on follow-up with the neurologist, the patient's wife reported that the patient's memory had significantly improved, although he intermittently would forget small details or events that happened one day back. No improvement was reported for the patient's anxiety.

On Day 32, a second routine EEG performed in the awake and asleep states showed during drowsiness an increased emergence of theta and delta rhythms and during sleep showed vertex sharp transients and K-complexes. The clinical interpretation of the EEG was that it showed abnormal results with mild slowing of the posterior dominant rhythm consistent with mild encephalopathy.

On Day 33, paraneoplastic and autoimmune CSF antibody panels returned negative for all antibodies. Specifically, the patient's spinal fluid was sent to Mayo Clinic Laboratories, Rochester, for the encephalopathy, autoimmune evaluation, and spinal fluid (Test ID: ENC2) which tests for AMPA-R Ab CBA, CSF; amphiphysin Ab, CSF; anti-glial nuclear Ab, type 1; anti-neuronal nuclear Ab, type 1; anti-neuronal nuclear Ab, type 2; anti-neuronal nuclear Ab, type 3; CASPR2-IgG CBA, CSF; CRMP-5-IgG, CSF; DPPX Ab IFA, CSF; GABA-B-R Ab CBA, CSF; GAD65 Ab assay, CSF; GFAP IFA, CSF; LGI1-IgG CBA, CSF; mGluR1 Ab IFA, CSF; NMDAR Ab CBA, CSF; Purkinje cell cytoplasmic Ab type Tr; Purkinje cell cytoplasmic Ab type 1; Purkinje cell cytoplasmic Ab type 2.

On Day 46, the patient completed carboplatin/etoposide (cisplatin was discontinued due to kidney disfunction) concurrent with 45 Gy in 1.5 Gy fractions BID radiation.

On Day 96, repeat Brain MRI showed no bitemporal hyperintensities or other acute intracranial processes.

On Day 131, repeat serum encephalopathy autoantibody panel returned entirely negative for any autoantibodies.

On Day 133, complete neurological and cognitive exams showed no deficits. As per the family, he is significantly better, although he still forgets some important dates but can recall them properly when reminded.

On Day 151, the patient completed prophylactic cranial irradiation with 25 Gy/10fx.

On Day 178, the patient continued to remain fully functional and stable.

## 2. Discussion

Limbic encephalitis (LE) is a rare disorder characterized by rapidly progressive memory loss, acute onset dementia, and personality change. LE may be autoimmune, inflammatory, i.e., paraneoplastic or infectious in etiology. It typically involves components of the limbic system including the anteromedial temporal cortex, hippocampus, and amygdala.

There are multiple causes of rapidly progressive dementia including infectious and metabolic etiologies. The patient should be evaluated for common infectious causes of altered mentation including HSV, HIV, and Creutzfeldt-Jakob disease. Once other metabolic or nutritional deficiencies (e.g., TSH, niacin, thiamine, celiac disease, and alcohol abuse) have been excluded, one must consider the possibility of a paraneoplastic presentation of encephalopathy [1].

Although the etiology of LE is not well understood, the current hypothesis suggests that the body reacts against particular antigens expressed on tumor cells, resulting in antibodies that then cross-react with similar antigens expressed on cells in the limbic system. Anti-Hu Ab, also known as anti-ANNA1, is most closely associated with paraneoplastic LE in SCLC [2], while anti-NMDAR Ab is associated with teratomas [3].

The definition and diagnostic criteria of autoimmune limbic encephalitis are steadily evolving in recent decades as data accumulates. In 2000, Gultekin presented the original diagnostic criteria, which include all four of the following:

(a) Symptoms of short-term memory loss, seizures, or psychiatric symptoms
(b) <4 year between the onset of neurological symptoms and the cancer diagnosis
(c) Exclusion of metastasis, infection, metabolic and vitamin deficits, and stroke etiologies
(d) ≥1 of the following:
  - CSF with inflammatory abnormalities (pleocytosis, increased protein concentration)
  - Hyperintensities on MRI FLAIR or T2 in the uni or bilateral temporal lobes
  - EEG with epileptic or slow activity involving the temporal lobes focally [4]

Our patient meets each of the abovementioned criteria: (a) he presented with STM loss, (b) with only two days between the onset of neurological symptoms and the cancer diagnosis. (c) CT and MRI showed no evidence of stroke or metastasis. The patient was afebrile, without leukocytosis, and without focal neurological deficits beyond his STM loss. The CSF pathogen panel returned negative. EEG showed no evidence of CJD. (d) (i) CSF showed lymphocytic pleocytosis, (ii) bilateral medial temporal lobe hyperintensities were present (left more than right) on Axial FLAIR. However, the latter finding had some degree of ambiguity and disagreement on interpretation. (iii) Notably, a repeat EEG showed mild slowing of the posterior dominant rhythm. Some experts opine that slowing of any kind on EEG, taken together with other clinical and imaging findings, is supportive of limbic encephalitis [5].

In 2005, Graus and Saiz suggested a revised set of criteria which included laboratory detection of a paraneoplastic antibody. More recently, there is a growing consensus that antibody status should not be included in the mandatory diagnostic criteria of autoimmune or paraneoplastic limbic encephalitis. This is because several weeks may pass before antibody tests' results return and, importantly, absence of antibodies does not necessarily exclude an autoimmune origin (see discussion below). Rather, the diagnosis should be driven by clinical presentation, imaging studies, and exclusion of infectious etiologies through more rapid serum and CSF laboratory tests [5, 6].

In 2016, several experts suggested a revised set of diagnostic criteria for definitive autoimmune limbic encephalitis, which includes all four of the following:

1. Subacute onset (less than 3 months) of working memory deficits, seizures, or psychiatric symptoms
2. Bilateral MRI brain abnormalities on medial temporal lobes
3. At least one of the following: (a) CSF pleocytosis and (b) EEG with epileptic or slow wave activity in the temporal lobes
4. Reasonable exclusion of alternative causes

If one of the first three criteria is not met, a diagnosis of definite LE can be made only by the detection of antibodies against cell-surface, synaptic, or onconeural proteins [6].

These criteria were intended for an umbrella diagnosis of limbic encephalitis. However, studies by Dalmau et al. in 2007-2008 of anti-NMDA-receptor-positive LE suggest that this particular form of LE should be treated as a new subcategory with its own diagnostic criteria. Of 92 anti-NMDAR-Ab-positive patients with EEG monitoring, 77% showed slow delta or theta activity, generalized or in frontotemporal regions. Only 22 of 100 anti-NMDAR-positive patients showed increased signal in the medial temporal lobes [7]. These findings of Dalmau et al. suggest that unlike the diagnostic criteria for the umbrella LE disease entity in anti-NMDAR encephalitis, MRI abnormalities on medial temporal lobe abnormalities should be unnecessary for diagnosis.

Of note, anti-NMDAR Ab alone is not specific to limbic encephalitis as it also can be found in multiple sclerosis and neuromyelitis optica [8].

It is significant that unlike our elderly male patient positive for serum anti-NMDAR Abs with SCLC, anti-NMDAR Abs have been more frequently described in younger women presenting with ovarian teratomas and in young men with testicular germ cell tumors [9]. Furthermore, in the literature, PLE SCLC is closely linked to anti-Hu Abs, not anti-NMDAR Abs of our patient [2].

Ironically, in our patient with neurological symptoms, the CSF was negative for autoimmune antibodies, while serum was found to be positive for anti-NMDAR Abs. The reason for such findings is unclear. However, in general, approximately half of autoimmune encephalitis cases are found to be antibody negative. Reasons for these frequent negative findings may include insensitivity of current clinical antibody testing, false-negative results caused by an insufficient quantity of antibody, and other technical issues such as antigen denaturation during tissue fixation. Furthermore, as in recent years, many new CNS autoantibodies have been discovered (e.g., anti-NMDAR, anti-LGI1, anti-Caspr2, and anti-GABAB receptor), and it is plausible that CNS inflammation in LE in some cases is caused by unknown or newly discovered autoantibodies that are not yet available for laboratory testing. In addition, there is a possibility that the temporal lobe inflammation is not antibody mediated [5, 8].

The goal of PLE therapy is to decrease the presentation of the antigen-expressing tumor cells, thereby limiting the number of cross-reactive antibodies. This goal is accomplished by tumor resection, radiation, chemotherapy, and immunosuppressive therapies [10]. In the study of Dalmau et al., at median 17-month follow-up, 47% attained full recovery, 46% remained with neuropsychiatric deficits, and 7% died from the disease—with treatments including tumor resection, intravenous immunoglobulin, plasma exchange, corticosteroids, rituximab, cyclophosphamide, and azathioprine [7] Patients typically do not have good response to first-line therapy. Our patient received only a single dose of one gram of solumedrol prior to decision to discontinue due to significant improvement with initiation of chemotherapy (cisplatin and etoposide). Not only did the patient have a remarkably good response without the need for immunotherapy but also improvement was noted almost immediately following the administration of chemotherapy. Such a response is not typically anticipated and noteworthy in this case for 3 reasons. Firstly, drastic improvement was seen despite this patient not being of the typical age group (young, female). Secondly, the presenting malignancy was atypical to the corresponding autoantibody (small-cell lung cancer which is more tightly associated with anti-Hu antibodies and present in elderly men). Thirdly, there was no CSF detection of the NMDAR antibody which is unusual but possible in the proper clinical context, and it was the likely diagnosis in this case as patient had significant response to a decrease in antigen burden once chemotherapy was initiated. Our patient with SCLC showed significant improvement in neurological status after chemotherapy and radiation. He remained stable at 6-month follow-up.

## 3. Conclusion

Our case brings attention to anti-NMDAR SCLC PLE, an uncommon disease entity. It is clear that the understanding of PLE, its subcategories, and accurate diagnostic criteria are continuously evolving as more data are reported and studied.

## References

[1] I. W. Altabakhi, H. M. Babiker, Paraneoplastic limbic encephalitis-StatPearls-NCBI bookshelf, https://www.ncbi.nlm.nih.gov/books/NBK519523/30137808

[2] Senties-Madrid H., Vega-Boada F. (2001). Paraneoplastic syndromes associated with anti-Hu antibodies. *The Israel Medical Association Journal : IMAJ*.

[3] Braverman J. A., Marcus C., Garg R. (2015). Anti-NMDA-receptor encephalitis: a neuropsychiatric syndrome associated with ovarian teratoma. *Gynecologic Oncology Reports*.

[4] Gultekin S. H. (2000). Paraneoplastic limbic encephalitis: neurological symptoms, immunological findings and tumour association in 50 patients. *Brain*.

[5] Ganesh A., Wesley S. F. (2018). Practice Current: when do you suspect autoimmune encephalitis and what is the role of antibody testing?. *Neurology: Clinical Practice*.

[6] Graus F., Titulaer M. J., Balu R. (2016). A clinical approach to diagnosis of autoimmune encephalitis. *The Lancet Neurology*.

[7] Dalmau J., Gleichman A. J., Hughes E. G. (2008). Anti-NMDA-receptor encephalitis: case series and analysis of the effects of antibodies. *The Lancet Neurology*.

[8] Lee S. K., Lee S.-T. (2016). The laboratory diagnosis of autoimmune encephalitis. *Journal of Epilepsy Research*.

[9] Boangher S., Mespouille P., Filip C.-M., Goffette S. (2016). Small-cell lung cancer with positive anti-NMDAR and anti-AMPAR antibodies paraneoplastic limbic encephalitis. *Case Reports in Neurological Medicine*.

[10] Shen K., Xu Y., Guan H. (2018). Paraneoplastic limbic encephalitis associated with lung cancer. *Scientific Reports*.

